# Geographic Variation in Morphology and Physiology of *Meretrix meretrix* (Linnaeus, 1758) Along the Chinese Coast

**DOI:** 10.3390/ani16010065

**Published:** 2025-12-25

**Authors:** Jinmeng Bao, Yue Zhu, Longyu Liu, Shuai Han, Fengbiao Wang, Haopeng Hu, Xuan Zhang, Lei Li, Mei Jiang

**Affiliations:** 1 College of Marine Science and Environment Engineering, Dalian Ocean University, Dalian 116023, China; 16621654437@163.com (J.B.); liulongyu1219@163.com (L.L.); 2 East China Sea Fisheries Research Institute, Chinese Academy of Fisheries Sciences, Shanghai 200090, China; alixyue@yeah.net (Y.Z.); 17634448779@163.com (S.H.); wangfengbiao1998@163.com (F.W.); hu15231265111@163.com (H.H.); zhangxuan@ecsf.ac.cn (X.Z.)

**Keywords:** *Meretrix meretrix* (Linnaeus, 1758), morphological variation, filtration rate, oxygen consumption rate, burrowing ability

## Abstract

The clam *Meretrix meretrix* (Linnaeus, 1758) is a commercially significant bivalve species widely distributed along China’s coast. To investigate the differences among geoducks from different geographic populations, this study investigated the geographical variations in morphology and key physiological traits among three populations spanning a latitudinal gradient: Liaoning Dandong (north), Jiangsu Rudong (center), and Guangxi Qinzhou (south). We analyzed nine morphological traits and measured physiological indicators—including filtration rate, feeding rate, oxygen consumption, ammonia excretion, and burrowing behavior—at two temperatures (18 °C and 20 °C). The results revealed significant morphological differentiation among the populations. Physiologically, the Guangxi population demonstrated superior adaptation to warmer conditions, exhibiting the highest feeding and metabolic rates at 20 °C. In contrast, the Liaoning population performed better at the lower temperature of 18 °C, while the Jiangsu population showed intermediate characteristics. Furthermore, burrowing capacity varied significantly, with the southern population having the highest burrowing rate. These findings demonstrate clear population-specific adaptations to local thermal environments, likely driven by long-term acclimatization. This study provides a crucial scientific basis for the conservation of genetic resources and informs regionalized aquaculture strategies for *M. meretrix*, emphasizing the importance of selecting locally adapted populations for cultivation.

## Simple Summary

The clam *Meretrix meretrix* (Linnaeus, 1758) is an important commercial seafood species along the coast of China. This study investigated how clams from three geographic populations spanning a latitudinal gradient—Liaoning Dandong (north), Jiangsu Rudong (center), and Guangxi Qinzhou (south)—differ in their shell morphology and key physiological functions. We found clear differences in shell shape and in processes such as feeding, metabolism, and burrowing behavior. Clams from the warmer southern region (Guangxi) performed better at a higher temperature (20 °C), showing higher feeding and metabolic rates. In contrast, clams from the cooler northern region (Liaoning) were more active at a lower temperature (18 °C). The Jiangsu population showed intermediate characteristics. These results demonstrate that the clams have developed population-specific adaptations to their local thermal environments. Our findings provide a scientific basis for selecting suitable clams for aquaculture in different regions and for conserving their genetic resources.

## 1. Introduction

*Meretrix meretrix* (Linnaeus, 1758) belongs to the phylum *Mollusca*, class *Lamellibranchia*, subclass *Heterodonta*, family *Veneridae*, genus *Meretrix*. Commonly known as the flower clam or sea clam, it inhabits soft intertidal zones along coastal areas and is a widely distributed, economically important bivalve in China’s coastal waters. Renowned for its delicious meat and rich nutritional value, it is also a traditional marine shellfish used in Chinese medicine [1]. Due to the extensive geographic distribution of populations across different regions, they may exhibit significant variations in morphological characteristics and physiological–ecological traits. These differences not only reflect their environmental adaptability but also provide a crucial scientific basis for germplasm resource conservation and artificial cultivation of *M. meretrix* [2]. In recent years, *M. meretrix* aquaculture has faced challenges such as germplasm degradation and slow growth rates. Research indicates that significant variations exist among geographically distinct *M. meretrix* populations in growth rates, reproductive capacity, and stress resistance, potentially closely related to local environmental factors such as water temperature, salinity, and feed availability [3,4].

Morphological variations serve as key indicators for distinguishing species and populations, enabling the identification of species. Morphological characteristics of *M. meretrix*, such as shell length, shell width, and body weight, are crucial indicators of its growth and development [5,6], significantly influencing both its live weight and carbon–nitrogen content [7,8]. These traits also function as vital markers for differentiating the clam’s provenance [9,10]. Observing and measuring the morphological characteristics of *M. meretrix* enables more effective selective breeding and screening of superior varieties. *M. meretrix* are filter-feeding mollusks primarily consuming microscopic phytoplankton, supplemented by protozoa, invertebrate larvae, and organic debris [11]. The filtration rate indicates the total volume of water filtered by filter-feeding mollusks per unit time, serving as a dynamic indicator of their feeding and physiological status. The feeding rate refers to the weight of particulate matter filtered by the mollusk per unit time. Studying both filtration and feeding rates aids in understanding the feeding habits of *M. meretrix*, optimizing feed selection and feeding practices in aquaculture. The oxygen consumption rate indicates the amount of oxygen consumed per unit body weight of the animal per unit time. It is a key indicator of an animal’s metabolic activity and can be used to assess *M. meretrix*’s energy metabolism level and environmental adaptability. Ammonia excretion rate denotes the amount of ammonia expelled by an organism through metabolic activity per unit time, reflecting protein metabolic intensity and energy utilization efficiency. In aquatic organisms such as shellfish, ammonia excretion rates are typically measured alongside oxygen consumption rates to assess their metabolic status and environmental adaptability. Simultaneous measurement of both parameters in the same individual allows calculation of the critical physiological–ecological index—the oxygen-to-nitrogen ratio (O:N Ratio)—a key indicator for determining energy substrate utilization. When environmental factors change (e.g., sudden temperature shifts, salinity fluctuations, dissolved oxygen decline, pH variations, pollutant exposure), animals must adapt by adjusting their physiological metabolism. Under thermal stress, altered metabolic enzyme activity may lead to abnormally elevated ammonia excretion rates and oxygen consumption rates. By comparing changes in ammonia excretion rates and O:N ratios before and after stress exposure, or across different adapted populations, one can quantitatively assess an organism’s tolerance to specific environmental pressures, its adaptive strategies, and the metabolic costs incurred. By measuring ammonia excretion rates and O:N ratios in farmed organisms fed diets with varying protein-to-fat ratios, one can scientifically evaluate feed energy utilization efficiency. This guides the development of environmentally friendly feeds that better align with animal metabolic needs while reducing nitrogen waste emissions. Selecting breeds that maintain low nitrogen excretion rates or exhibit higher energy utilization (high O:N ratios) under specific environmental conditions (e.g., high temperatures, low oxygen) facilitates the development of new breeds with enhanced stress resistance and growth efficiency. *M. meretrix* exhibits a burrowing lifestyle, relying on the retraction and extension of its foot to burrow into sand layers. This burrowing behavior represents a key adaptive strategy for clams responding to intertidal environmental fluctuations, progressing through three stages: preparation, burrowing, and completion [12]. Individuals of *M. meretrix* exhibit morphological variation, which also results in differences in their sand-burrowing depth [13]. Consequently, sand-burrowing capabilities vary among different populations.

Previous studies have examined morphological and physiological–ecological indicators of different geographic populations of *M. meretrix* [14,15]. This experiment selected three geographic populations from Liaoning Dandong, Jiangsu Rudong, and Guangxi Qinzhou, representing a north-to-south continuum of coastal populations in China, to fill research gaps. Additionally, this experiment simultaneously examined morphological indicators and four physiological indicators, enabling a direct observation of how temperature changes affect each indicator across different populations. Given the environmental temperature variations across China’s coastal latitudes, this study conducted morphological comparisons and physiological–ecological experiments on three geographic populations of *M. meretrix* from Liaoning Dandong, Jiangsu Rudong, and Guangxi Qinzhou. The core objective is to reveal adaptive differences in temperature tolerance among *M. meretrix* populations originating from different latitudes. By measuring key physiological indicators—including filtration rate, feeding rate, oxygen consumption rate, and ammonia excretion rate—under temperature gradients, we investigated whether adaptive differentiation correlates with the environmental history and genetic background of geographic origins. This research provides a scientific basis for germplasm conservation: selecting heat-tolerant superior populations for stress-resistant variety breeding, identifying sensitive populations for targeted conservation strategies, and guiding optimized aquaculture practices by planning regionalized farming layouts based on population-specific heat adaptation differences.

## 2. Materials and Methods

### 2.1. Materials

**Figure 1:**
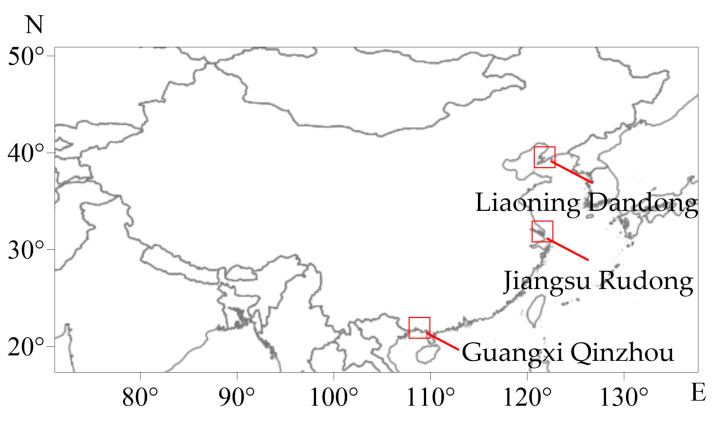
Schematic Diagram of Sampling Locations.

The *M. meretrix* populations used in this experiment were collected from three regions: Liaoning Dandong (E 124°23′, N 40°7′, Yellow Sea); Jiangsu Rudong (E 121°18′, N 32°33′, East China Sea); and Guangxi Qinzhou (E 108°39′, N 21°56′, South China Sea) (Figure 1). They were transported to the laboratory under low-temperature conditions and temporarily reared in artificial seawater at room temperature. Salinity was gradually adjusted to the optimal survival salinity for clams, set at 20 ± 0.5‰ for this experiment. They were fed twice daily with *Platymonas helgolandica* (C.K. Tseng, T.J. Chang, 1964). Thirty individuals of similar size (35~45 mm) and age (2-year-old) were selected from each population as experimental materials.

### 2.2. Methods

#### 2.2.1. Morphological Measurements

**Figure 2:**
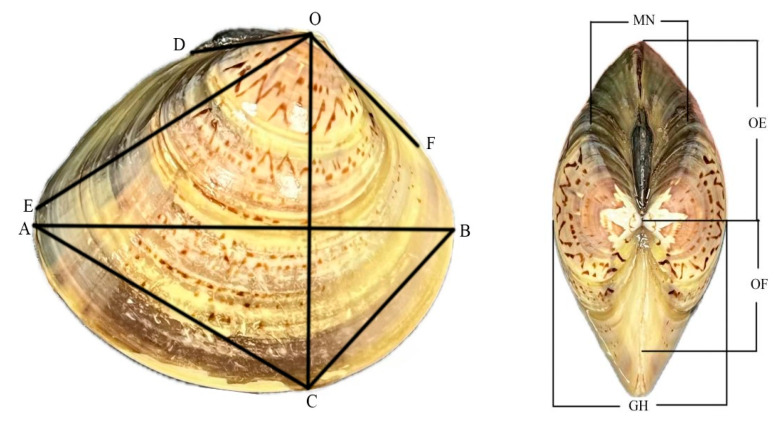
Morphological measurement sites of *Meretrix meretrix* (Linnaeus, 1758). Nine measurable traits: shell length AB; shell width GH; shell height OC; ligament length OD; projection length OE; projection width MN; hinge length OF; anterior–ventral margin distance BC; posterior–ventral margin distance AC.

Thirty individuals of similar size were randomly selected from each of the three populations. Nine measurable traits were assessed using a vernier caliper (accurate to 0.01 mm) as shown in Figure 2. Dry weight and shell weight were measured using an electronic balance (Sartorius, Göttingen, Germany, accurate to 0.01 g) to calculate the fatness index.

#### 2.2.2. Physiological and Ecological Indicator Measurements

Sourced from a coastal area with an annual mean temperature range of 11–24 °C, the clams were subjected to two elevated temperature treatments in the experiment: 18 °C (+1 °C above ambient) and 20 °C (+3 °C above ambient). The selected temperatures (18 °C and 20 °C) fall within the optimal growth range of *M. meretrix.* A 2 °C difference was sufficient to elicit observable changes in physiological metabolism without inducing stress responses. For each region, thirty clams were allocated into three parallel treatment groups and one blank control group. After temperature adjustment using heating rods, the clams were transferred to 10 L glass tanks. An appropriate volume of *Platymonas subcordiformis* suspension was added to each tank to maintain a constant algal concentration of 2 × 10^5^ cells/mL throughout the experiment, ensuring normal feeding activity. Algal concentration was monitored by measuring absorbance and converting it to cell density using a pre-established standard curve. Prior to the experiment, GF/C glass fiber filter papers (pore size 1.2 μm) were ashed at 450 °C for 6 h and labeled. To determine particulate organic matter (*POM*), 500 mL of the water sample was filtered through a prepared GF/C filter. The filter was rinsed with 0.5 mol/L ammonium formate (≈10 mL) to remove salts, dried at 110 °C to constant weight (*m*_110_), and then ashed at 450 °C for 6 h before reweighing (*m*_450_). *POM* was calculated as *mPOM* = *m*_110_ − *m*_450_. The experiment lasted for 2 h and was conducted during normal daytime hours. Suspended algal biomass (mg), dissolved oxygen (mg/L), and ammonia-nitrogen (μg/mL) were measured in each beaker before and after the incubation. Suspended algae were quantified by filtering 500 mL of water, followed by drying and weighing the residue. Dissolved oxygen was measured with an oxygen meter, and ammonia–nitrogen was determined spectrophotometrically using the salicylic acid method. Following the experiment, clam soft tissues were dissected and dried at 65 °C for subsequent analysis. To eliminate oxygen exchange between the atmosphere and the water, all experimental tanks were completely sealed during all oxygen consumption rate determination experiments.

Filtration rate calculation formula:

$$CR=\frac{V\times \ln\left(c_{0}/c_{t}\right)}{\left(W\times t\right)}$$

In the above formula, *V* represents the volume of liquid in the experimental tank (L), *W* denotes the dry weight of the soft body of the clam (g), and *c*_0_ and *ct* are the concentrations of *POM* (particulate organic matter) in the experimental water at the start of the experiment and at time *t*, respectively. The *POM* method is determined in the same manner as the filtration rate.

Feeding rate calculation formula:

$$IR=\frac{V(c_{0}-c_{t})}{\left(W\times t\right)}$$

In the above formula, *V* represents the volume of liquid in the experimental tank (L), *W* denotes the dry weight of the soft body of the clam (g), and *c*_0_ and *ct* are the concentrations of *POM* in the experimental water at the start of the experiment and at time *t*, respectively.

Oxygen consumption rate calculation formula:

$$OR=\frac{(DO_{0}-DO_{t})V}{WT}\times 100\%$$

In the above formula, *DO*_0_ represents the initial oxygen content, *DO_t_* represents the final oxygen content, *V* represents the volume of liquid in the experimental tank (L), *W* denotes the dry weight of the soft body of the clam (g), and *T* denotes the duration of the experiment.

Ammonia removal rate calculation formula:

$$NR=\frac{(N_{t}-N_{0})V}{WT}\times 100\%$$

In this formula, *N*_0_ is the initial ammonia nitrogen content, *N_t_* is the final ammonia nitrogen content, *V* is the water volume (L), *W* is the dry weight of the soft body of the clam (g), and *T* is the duration of the experiment.

Place 30 individuals of each of the three geographic populations of *M. meretrix* in a water tank filled with 20 cm of sea sand, allowing them to be exposed on the surface of the sea sand at room temperature (the natural substrate in all three regions is predominantly sandy; to ensure consistent experimental conditions, the sea sand was sourced from Qidong, Jiangsu Province). Burrowing depth was observed every 8 h over 24 h, with measurements recorded.

Formula for calculating sand burial rate:

$$P=\frac{N_{t}}{N_{0}}\times 100\%$$

In this formula, *P* represents the sand burial rate (%), and *N_t_* and *N*_0_ denote the number of completely buried individuals (grains) and the total number of test individuals (grains), respectively.

### 2.3. Data Processing and Analysis

Experimental data were analyzed using Excel (Microsoft Corporation, Redmond, WA, USA), SPSS 27.0 (IBM, Armonk, New York, USA), and Origin 2024 (OriginLab, Northampton, MA, USA). SPSS 27.0 software was employed to perform one-way ANOVA, principal component analysis (PCA), and cluster analysis on morphological data from the three populations of *M. meretrix*. For cluster analysis and PCA, to eliminate the influence of individual size variation on morphometric parameters, the ratios of each trait index to shell length were used: shell height/shell length, shell width/shell length, shell length, ligament length/shell length, projection length/shell length, projection width/shell length, hinge length/shell length, anterior–ventral margin distance/shell length, posterior–ventral margin distance/shell length, and total mass/shell length. Principal component analysis (PCA) is a dimensionality reduction technique that transforms correlated variables into uncorrelated principal components through orthogonal transformation, retaining most of the original data’s information with a few composite indicators [16]. In this study, multiple morphological indicators of the *M. meretrix* were processed via PCA to generate two composite indicators, ensuring independence among principal components and resolving interference from correlations between morphological indicators. Cluster analysis is an unsupervised learning method for exploring intrinsic grouping structures within data, with core tasks including similarity metric calculation and noise resistance processing [17]. This study examined morphological similarity among geographically distinct populations of *M. meretrix*. The shortest Euclidean distance method was used to calculate morphological differences between samples, followed by systematic clustering to classify clam samples into distinct groups. SPSS 27.0 software was used for significance analysis and two-way ANOVA of physiological and ecological indicators, while Origin 2024 software was employed for plotting experimental results. Excel was utilized to calculate the sand-burrowing rate and perform one-way ANOVA on burrowing capacity, with a significance level set at 0.05.

## 3. Results

### 3.1. Differences in Morphological Traits Among Geographically Distinct Meretrix meretrix (Linnaeus, 1758) Populations

**Figure 3:**
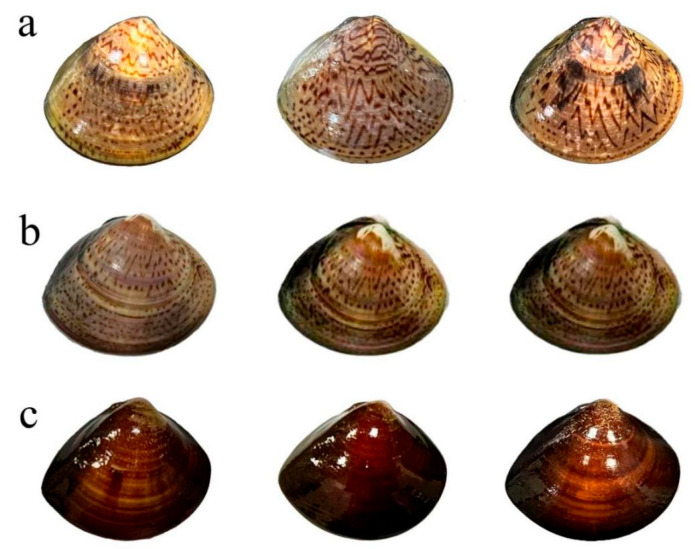
External morphology of *Meretrix meretrix* (Linnaeus, 1758) from Liaoning Dandong (**a**), Jiangsu Rudong (**b**), and Guangxi Qinzhou (**c**).

In this experiment, we collected populations of *M. meretrix* from three geographic regions: Liaoning Dandong, Jiangsu Rudong, and Guangxi Qinzhou. Nine measurable traits, along with wet weight and dry weight, were assessed. The external morphology of the three geographic populations is shown in Figure 3. Differences in shell color and patterns were observed among the three populations: the Dandong population exhibited a light yellowish-brown color with distinct patterns extending from the top toward the edges; The Rudong population exhibited paler patterns and darker shell coloration; the Qinzhou population displayed the darkest shell color, appearing deep brown with minimal patterns. Measurement results and significance analysis for morphological traits across the three populations are presented in Table 1, with trait values expressed as mean ± standard deviation. The analysis reveals significant variations in traits across the three populations. Among the nine measurable traits, the coefficient of variation for ligament length (OD) was highest in the Liaoning Dandong population (7.98%), the coefficient of variation for minor hinge length (OF) was highest in the Jiangsu Rudong population (12.72%), while the Guangxi Qinzhou population exhibited the highest coefficient of variation for projection width (MN) at 13.58% (>10%). This indicates a significant difference in mantle width between the Qinzhou population and the other two groups. The coefficients of variation for body weight exceeded 10% for all three populations, confirming significant differences in body weight among them. The Liaoning Dandong population and the Guangxi Qinzhou population were morphologically highly similar, showing significant differences (*p* < 0.05) from the Jiangsu Rudong population in six traits: shell length, shell height, shell width, body weight, posterior–ventral margin distance (BC), and ligament length (OD). Among these, the Dandong and Qinzhou populations had higher average values for shell length, shell height, shell width, and body weight than the Rudong population, while their average values for posterior–ventral margin distance (BC) and ligament length were lower than those of the Rudong population. Significant differences were observed between the Liaoning Dandong and Guangxi Qinzhou populations in three traits: anterior–ventral margin distance (AC), hinge length (OF), and projection width (MN). The Liaoning Dandong population exhibited a higher average AC than both the Rudong and Qinzhou populations. For OF, the average values were as follows: Liaoning Dandong > Jiangsu Rudong > Guangxi Qinzhou. The Qinzhou group exhibited the smallest average MN, significantly differing from the other two groups. The projection length (OE) of all three groups was approximately 30 mm, showing no significant differences.

**Figure 4:**
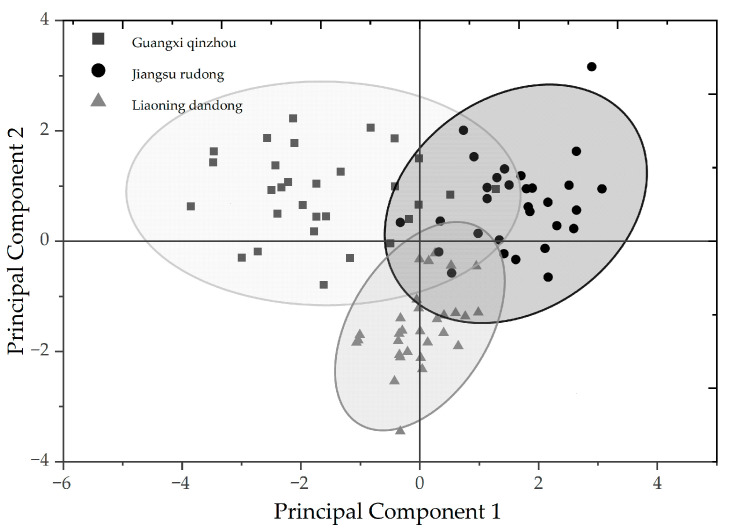
Scatterplot of principal component analysis for morphological traits of three geographic populations of *Meretrix meretrix* (Linnaeus, 1758).

This study used principal component analysis (PCA) to analyze morphological differences in samples from three geographic populations: Guangxi Qinzhou, Jiangsu Rudong, and Liaoning Dandong. Figure 4 is a scatter plot of the principal component analysis. Three principal components were extracted, each with an eigenvalue greater than 1, and the cumulative contribution rate was 61.238%, which can well reflect the main variation information of shell morphology, indicating that the complexity of shell morphology can be effectively summarized by a limited number of comprehensive traits. As shown in Table 2, among them, Principal Component 1 (shell transverse development) is the main factor causing geographic population differences, while Principal Component 2 mainly represents the longitudinal structural characteristics of the shell. Principal Component 3 mainly reflects the thickness and weight of the shell.

**Figure 5:**
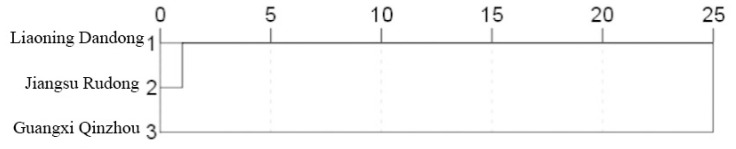
Cluster analysis of three geographic populations.

Using the EuclideanDistance, a cluster analysis was conducted on nine measurable traits across three distinct geographic populations of *M. meretrix*. The results, shown in Figure 5, indicate that the three populations clustered into a single group. Among them, the Jiangsu Rudong population and the Liaoning Dandong population exhibited closer distances and clustered together first. This indicates that the Liaoning Dandong and Jiangsu Rudong populations exhibit greater morphological similarity, while the Guangxi Qinzhou population shows more pronounced morphological differences from both groups, reflecting a higher degree of divergence.

### 3.2. Differences in Physiological and Ecological Indicators of Three Meretrix meretrix (Linnaeus, 1758) Populations at Different Temperatures

#### 3.2.1. Differences in Filtration Rates Among Three Geographically Distinct Populations of *Meretrix meretrix* (Linnaeus, 1758) at Different Temperatures

**Figure 6:**
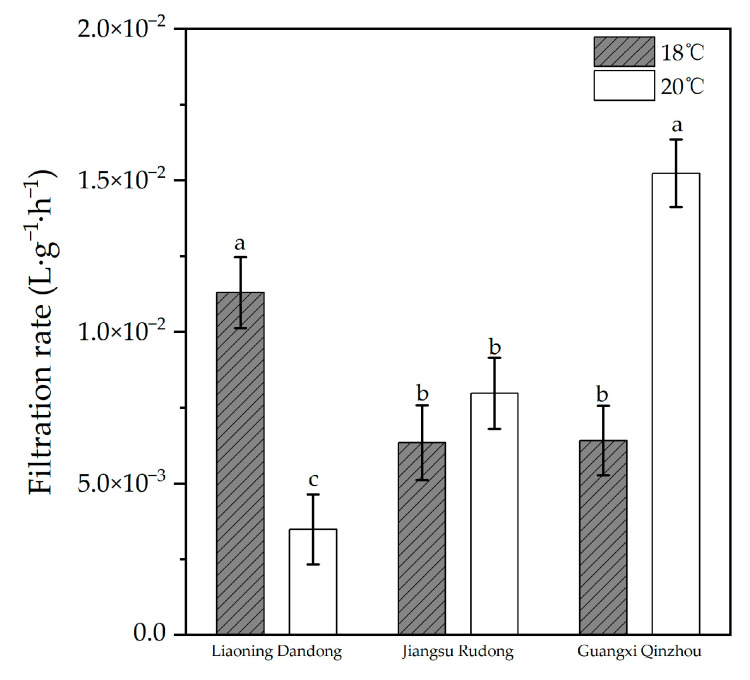
Filtration rates of three geographic populations at different temperatures. (The letter labels on the bar chart (such as a, b, c) are based on the results of statistical tests: groups with the same letter are not significantly different from each other, while groups with different letters are significantly different).

The filter rate results for the three geographic populations of *M. meretrix* under different temperatures are shown in Figure 6. The water filtration rate of each clam species varied with temperature. A two-way ANOVA comparing the filtration rates of clams from Liaoning Dandong, Jiangsu Rudong, and Guangxi Qinzhou revealed significant interactions between region and temperature (*p* < 0.05). This indicates that clams from different regions exhibit distinct response patterns to temperature changes. Single-factor ANOVA of filter rates at the same temperature revealed significant differences among the three populations at 18 °C (*p* < 0.05), with Liaoning Dandong > Jiangsu Rudong > Guangxi Qinzhou. At 20 °C, the clams from Guangxi Qinzhou exhibited the highest values and showed the greatest significance among the three populations, with the significance order being Guangxi Qinzhou > Jiangsu Rudong > Liaoning Dandong.

#### 3.2.2. Differences in Feeding Rates Among Three Geographically Distinct Populations at Different Temperatures

**Figure 7:**
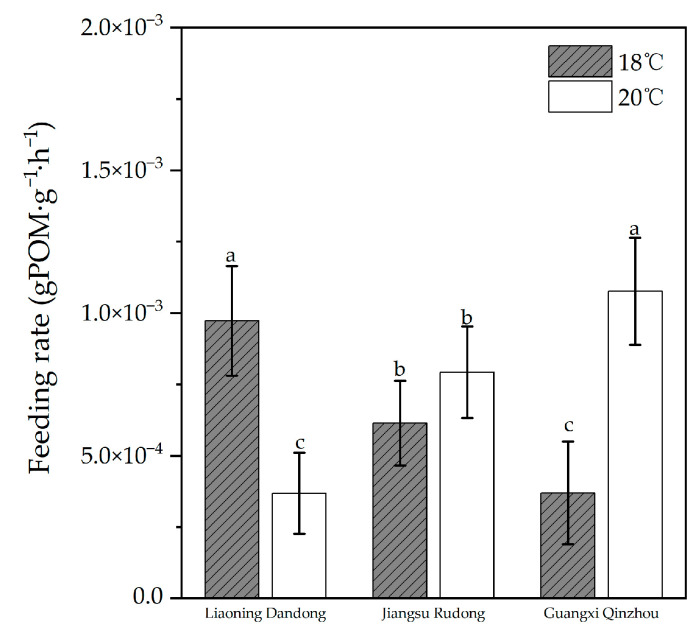
Feeding rates of three geographic populations at different temperatures. (The letter labels on the bar chart (such as a, b, c) are based on the results of statistical tests: groups with the same letter are not significantly different from each other, while groups with different letters are significantly different).

Figure 7 shows the feeding rate indicators for the three geographic populations of *M. meretrix* at different temperatures. The two-way ANOVA results indicate significant differences in feeding rates among clam populations from different regions at different temperatures (*p* < 0.05), with a significant interaction effect between region and temperature (*p* < 0.05). The feeding rates of all three clam populations responded to different temperatures, exhibiting significant differences between groups. At 18 °C, the Liaoning Dandong population showed the highest feeding rate, while the Guangxi Qinzhou population had the lowest. At 20 °C, the Guangxi Qinzhou population exhibited the highest feeding rate among the three groups, and its feeding rate at 20 °C was higher than at 18 °C, consistent with the results for water filtration rates.

#### 3.2.3. Differences in Oxygen Consumption Rate and Ammonia Excretion Rate Among Three Geographically Distinct Populations at Different Temperatures

**Figure 8:**
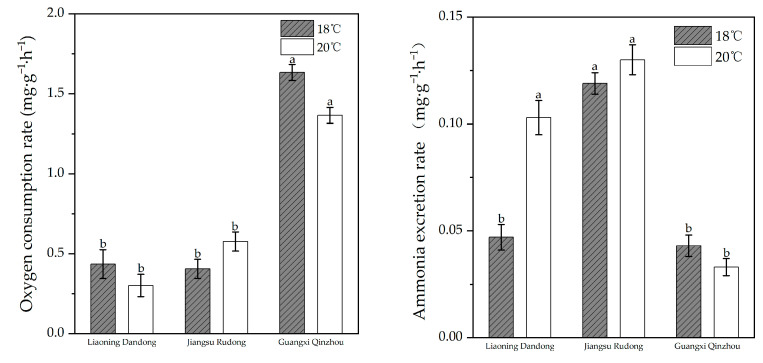
Oxygen consumption rate and ammonia excretion rate of three geographic populations at different temperatures. (The letter labels on the bar chart (such as a, b, c) are based on the results of statistical tests: groups with the same letter are not significantly different from each other, while groups with different letters are significantly different).

The oxygen consumption and ammonia excretion rates of different geographical populations of *M. meretrix* at 18 °C and 20 °C are shown in Figure 8. Two-way ANOVA revealed a significant interaction effect between geographical population and temperature (*p* < 0.05). At 18 °C, the Guangxi Qinzhou population exhibited the highest oxygen consumption rate (1.63 ± 0.05 mg/g^−1^·h^−1^), significantly higher than the Liaoning Dandong population (0.44 ± 0.09 mg/g^−1^·h^−1^) and the Jiangsu Rudong population (0.41 ± 0.06 mg/g^−1^·h^−1^). When the temperature rose to 20 °C, the oxygen consumption rate of the Rudong group increased (0.58 ± 0.06 mg/g^−1^·h^−1^), yet remained lower than that of the Qinzhou group (1.37 ± 0.05 mg/g^−1^·h^−1^). Analysis of ammonia excretion rates revealed that the Jiangsu Rudong group maintained the highest rates at both temperatures (0.12 ± 0.005 mg/g^−1^·h^−1^) and 0.13 ± 0.007 mg/g^−1^·h^−1^), while the Guangxi Qinzhou group exhibited the lowest rates (0.04 ± 0.005 mg/g^−1^·h^−1^ and 0.03 ± 0.004 mg/g^−1^·h^−1^), and 0.03 ± 0.004 mg/g^−1^·h^−1^). The oxygen consumption rate of the Liaoning Dandong population increased at 20 °C but remained lower than that of the Jiangsu Rudong population. Table 3 shows the O:N ratios of the three populations at 18 °C and 20 °C. Data indicate that the Liaoning Dandong population exhibited a higher ratio at 18 °C, while both the Jiangsu Rudong and Guangxi Qinzhou populations demonstrated higher oxygen-to-nitrogen ratios at 20 °C compared to their values at 18 °C.

#### 3.2.4. Differences in Sand Burrowing Capabilities Among Three Geographically Isolated Populations of *Meretrix meretrix* (Linnaeus, 1758)

**Figure 9:**
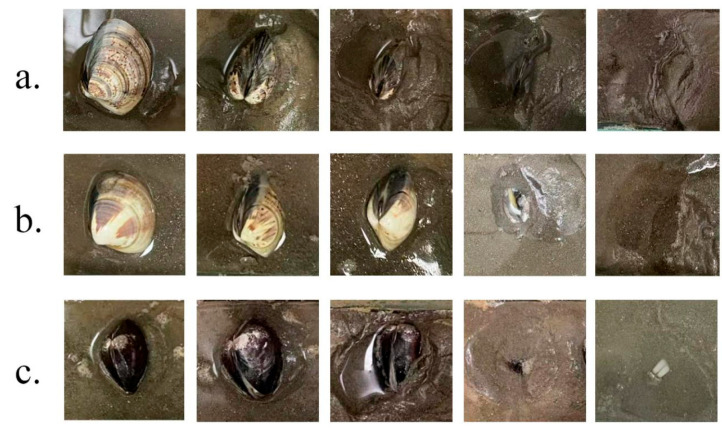
Sand-burrowing process of *Meretrix meretrix* (Linnaeus, 1758) (**a**) Liaoning Dandong population; (**b**) Jiangsu Rudong population; (**c**) Guangxi Qinzhou population.

During the burrowing process, *M. meretrix* species first extend their siphons and foot, then perform vertical shell movement and downward diving. After burrowing into the substrate, they leave their siphons on the surface to complete the process. This study compared the burrowing depth and burrowing rate among three geographic populations under identical substrate conditions, revealing significant differences between populations (*p* < 0.05). Figure 9 illustrates the burrowing process of the three geographic populations. Table 4 presents the burrowing depth and rate for each population. The Guangxi Qinzhou population exhibited the highest burrowing rate (65%), significantly exceeding those of Liaoning Dandong and Jiangsu Rudong. However, the Jiangsu Rudong population achieved the greatest average burrowing depth (5.36 ± 0.36 cm), while the Liaoning Dandong population recorded the shallowest burrowing depth among the three.

## 4. Discussion

### 4.1. Morphological Traits Among Geographically Distinct Clam Populations

Measurement data and significance analysis results indicate that the three populations exhibit significant differences across all nine quantifiable trait indices. Under varying geographical habitats, the phenotypic traits of the same species may differ [18,19]. Such morphological traits likely stem from differing habitat conditions caused by geographical disparities [20]. Bergmann’s Rule demonstrates the influence of climate on biological traits [21]. As an intertidal mollusk, differences in the survival environment of *M. meretrix*—such as temperature, salinity, and food availability—inevitably lead to variations in phenotypic traits. For example, the Rudong population in Jiangsu exhibits the widest shell width, potentially linked to its historical environment in the East China Sea. For example, waves in the East China Sea may influence the shell morphology of *M. meretrix*, resulting in a flatter shape [22]. The Guangxi Qinzhou population exhibits the highest coefficient of variation in projection width, reflecting its unique adaptation to the highly disturbed South China Sea environment. Principal component analysis indicates that Principal Component 1 correlates with hinge development and anterior shell growth bias, while Principal Component 2 negatively correlates with hinge length, further confirming the link between morphological traits and functional adaptations. Geographically, the three populations exhibit distinct spatial distributions and varying environmental conditions. Liaoning Dandong borders the northern Yellow Sea, characterized by low annual average water temperatures with minimal seasonal variation. Jiangsu Rudong lies adjacent to the East China Sea, while Guangxi Qinzhou borders the South China Sea. Both the East China Sea and South China Sea exhibit pronounced seasonal water temperature fluctuations, with higher summer temperatures in the East China Sea and even warmer surface temperatures in the South China Sea during summer [23,24,25]. Additionally, distinct selective signals on the genetic genomes of different populations can lead to phenotypic variations [18,26]. Thus, the morphological polymorphism of the *M. meretrix* is influenced by both environmental and genetic factors. In aquaculture and breeding, optimizing shellfish growth performance and economic traits can be achieved by regulating the environment or selecting individuals with specific shell morphologies [27].

### 4.2. Differences in Physiological and Ecological Indicators of Three Clam Species at Different Temperatures

Temperature is a critical factor in shellfish aquaculture. As a poikilothermic species, the survival environment of *M. meretrix* not only influences its phenotypic traits but also affects its physiological and ecological indicators, leading to variations [28,29]. The annual average water temperature of the Yellow Sea ranges from 15 to 24 °C, while that of the East China Sea is approximately 20 to 24 °C. The South China Sea maintains an average annual water temperature of around 22 °C. Salinity levels across these three sea areas show little variation. The preferred substrate type for *M. meretrix* survival is silty-sandy sediment. The annual average temperature range in the marine areas where the selected clams were sourced was 11~24 °C. This study compared differences in water filtration rate, feeding rate, oxygen consumption rate, and ammonia excretion rate among three geographically distinct populations of clams under two temperatures (18 °C and 20 °C). Results revealed significant differences in temperature response patterns among the three populations: the Liaoning Dandong population exhibited higher activity at lower temperatures (18 °C), while the Rudong population showed markedly increased water filtration and feeding rates with rising temperatures, though still lower than the Guangxi Qinzhou population. Inter-population differences in water filtration and feeding rates were statistically significant at *p* < 0.05. The Dandong population exhibited negative correlations between water filtration and feeding rates with increasing temperature, whereas the Rudong and Qinzhou populations showed positive correlations. Notably, the Qinzhou population demonstrated significantly higher filtration and feeding rates at 20 °C than the other populations, indicating superior adaptation to high-temperature environments. Research indicates that the filtration rate of bivalve mollusks is influenced by environmental parameters, with the common clam responding to temperature changes through behavioral and physiological adaptations [30,31]. Rising temperatures reduce water kinematic viscosity, thereby decreasing viscous resistance in the gill canal system and lowering filtration velocity [32]. Both filtration rate and feeding rate are related to the filter-feeding capacity of shellfish. Therefore, both are influenced by temperature. Within the optimal temperature range, filtration rate and feeding rate increase with rising temperature, but decline when temperatures exceed the optimal range [33].

At the same temperature, oxygen consumption and ammonia excretion rates differ significantly (*p* < 0.05) between different populations. Both oxygen consumption and ammonia excretion are important indicators of animal metabolism [34]. The beating frequency of cilia in bivalve gills is influenced by temperature, and changes in ciliary beating frequency alter oxygen consumption rates. Thus, temperature regulation of ciliary beating frequency indirectly affects bivalve oxygen consumption [35]. The oxygen consumption rate of the Guangxi Qinzhou population remained at its highest level under both temperatures, yet exhibited the lowest ammonia excretion rate. Combined with its exceptionally high O:N ratio (>30) [36], this indicates vigorous energy metabolism and high energy utilization efficiency in this population, primarily relying on fats and carbohydrates for energy—a metabolic strategy. The Liaoning Dandong population exhibited higher oxygen consumption at 18 °C, yet its O:N ratio decreased with rising temperatures, indicating more efficient metabolic balance maintenance at lower temperatures. The oxygen-to-nitrogen ratios across all three populations further suggest that the Dandong group is better adapted to cold environments, while the Qinzhou group thrives in warmer conditions.

Significant differences exist in physiological activities such as feeding, respiration, and excretion among geographically distinct populations of *M. meretrix*. These variations stem from the combined effects of habitat and genetic background [14,37]. Due to the large latitudinal span, the three populations inhabit marine areas with differing light conditions, directly influencing their water filtration and feeding behaviors [38]. Long-term habitat differences also lead to distinct microbial community compositions among populations, which in turn affect the clam’s immune and physiological functions [39,40]. This environmental heterogeneity promotes geographic isolation and genetic diversity, enabling different populations to evolve unique stress-resistant genes to adapt to environmental fluctuations [41,42,43].

This study compared three geographic populations under identical substrate conditions and found that the Guangxi Qinzhou population of *M. meretrix* exhibited the strongest sand-burrowing capacity. The clams rely on their well-developed adductor muscles for burrowing, a behavior serving as an adaptive strategy against environmental stresses such as water flow, predation, and desiccation. This behavior effectively prevents water loss and overheating [44]. The Qinzhou population exhibited the highest sand-burrowing rate (65%) and strongest response capacity, while the Rudong population reached the greatest burrowing depth (5.36 ± 0.36 cm), indicating a tendency for deep burrowing to evade predators. The Dandong population demonstrated lower values for both metrics. In natural environments, clams typically inhabit substrates at depths of 1~20 cm. When exposed, they must rapidly burrow downward to withstand threats from waves and turbulent currents. Differences in burrowing behavior among species or geographic populations stem from two primary categories: environmental factors and biological factors. Biological factors further encompass genetic influences and shell morphology. Environmental factors include ambient temperature and substrate type. Long-term exposure to differing habitats can alter genetic information, leading to variations in burrowing ability [45,46,47]. Furthermore, burrowing capacity is a critical determinant of intertidal mollusk distribution and survival, often constrained by morphology and body size [48]. For instance, the rounded and blunt-shaped clam fails to burrow effectively in harder substrates, whereas the streamlined clam achieves rapid burial [49]. Consequently, morphological differences and long-term environmental adaptations result in varying burrowing capabilities among the three clam groups.

The aforementioned studies indicate that differences among geographically distinct populations of *M. meretrix* manifest across multiple dimensions of morphology and physiological behavior. These variations are adaptations to long-term environmental conditions in their respective marine habitats, including water temperature, wave action, and food availability. Moreover, environmentally induced morphological differences influence physiological behavior, while physiological adaptations conversely lead to morphological variations. Under prolonged environmental pressures, genetic divergence emerges within clam populations, enabling them to adapt to their living environments.

## 5. Conclusions

(1) Morphological analysis revealed significant overall differences among the three populations (*p* < 0.05). The Jiangsu Rudong population exhibited the largest shell width (32.75 ± 2.06 mm), while the Guangxi Qinzhou population showed the highest coefficient of variation in projection width (13.58%), significantly differing from the Liaoning Dandong population.
(2) Physiological and ecological indicators also varied among the three populations: At 18 °C, the Liaoning Dandong population exhibited the highest water filtration rate and feeding rate, while the Guangxi Qinzhou population showed significant increases in both indicators at 20 °C. The oxygen consumption rate was highest in the Guangxi Qinzhou population, while the Jiangsu Rudong population showed a significant increase at 20 °C. The ammonia excretion rate remained consistently highest in the Jiangsu Rudong population.
(3) The three populations also exhibited differences on ability to burrow of the clam. The Guangxi Qinzhou population had the highest burrowing rate, while the Jiangsu Rudong population achieved the greatest burrowing depth.

## Figures and Tables

**Table 1:** Significance analysis results of morphological differences among different geographical populations of *Meretrix meretrix* (Linnaeus, 1758). Different letters marked on the same trait indicator in the table indicate significant differences (*p* < 0.05), while the same letters indicate that the differences are not significant (*p* > 0.05).

Characteristic	Liaoning Dandong		Jiangsu Rudong		Guangxi Qinzhou	
	Mean ± SD	Coefficient of Variation %	Mean ± SD	Coefficient of Variation %	Mean ± SD	Coefficient of Variation %
AB Shell length (mm)	40.967 ± 1.738 ^a^	4.24	38.843 ± 1.853 ^b^	4.77	40.763 ± 1.803 ^a^	4.42
AC Anterior-ventral margin distance (mm)	28.963 ± 1.599 ^a^	5.25	25.880 ± 2.776 ^b^	10.73	24.953 ± 2.073 ^b^	8.31
BC Posterior-ventral margin distance (mm)	21.294 ± 1.505 ^b^	7.07	22.777 ± 2.068 ^a^	9.08	21.177 ± 2.015 ^b^	9.52
OC Shell Height (mm)	34.607 ± 1.758 ^a^	5.08	19.397 ± 1.067 ^b^	5.50	34.340 ± 1.877 ^a^	5.47
OD Ligament length (mm)	10.320 ± 0.824 ^b^	7.98	11.277 ± 1.105 ^a^	9.80	10.533 ± 1.045 ^b^	9.92
OE Projection length (mm)	30.361 ± 1.680 ^a^	5.53	30.263 ± 1.715 ^a^	5.67	30.823 ± 2.057 ^a^	6.67
OF Hinge length (mm)	17.250 ± 1.091 ^a^	6.32	13.367 ± 1.701 ^b^	12.72	9.543 ± 0.863 ^c^	9.05
GH Shell Width (mm)	20.580 ± 0.887 ^a^	4.31	32.750 ± 2.059 ^b^	6.29	20.413 ± 0.837 ^a^	4.10
MN Shield Width (mm)	12.099 ± 0.674 ^a^	5.57	12.523 ± 1.071 ^a^	8.55	11.413 ± 1.550 ^b^	13.58
Body weight (g)	18.525 ± 2.216 ^a^	11.96	15.986 ± 2.132 ^b^	13.34	17.754 ± 1.943 ^a^	10.95
Dry weight (g)	5.37 ± 1.004	-	2.73 ± 0.194	-	4.87 ± 0.469	-
Fatness (%)	7.290	17.89	4.974	6.56	8.790	7.99
Shell width coefficient	0.357	-	0.350	-	0.367	-

**Table 2:** Principal component analysis loadings. Characteristic ratio represents the ratio of each trait index to shell length, thereby eliminating the influence of individual size variations within the sample on morphometric parameters.

Characteristic Ratio	Loadings		
	Principal Component 1	Principal Component 2	Principal Component 3
GH/AB	−0.177	0.163	0.811
OC/AB	0.337	−0.101	0.368
OD/AB	0.597	0.530	0.012
OE/AB	0.446	0.577	0.005
MN/AB	0.784	−0.093	−0.047
OF/AB	0.510	−0.690	0.127
AC/AB	0.630	0.495	0.179
BC/AB	0.487	−0.601	0.313
Total mass/AB	−0.614	0.171	0.461
Cumulative Contribution %	28.720	48.419	61.238

**Table 3:** O:N ratios of three geographic populations at different temperatures.

Region	Temperature	O:N
Liaoning Dandong	18 °C	9.28
	20 °C	2.94
Jiangsu Rudong	18 °C	3.42
	20 °C	4.44
Guangxi Qinzhou	18 °C	37.99
	20 °C	41.38

**Table 4:** Comparison of the burrowing depth and burrowing rate among three geographic populations. (The letter labels on the bar chart (such as a, b, c) are based on the results of statistical tests: groups with the same letter are not significantly different from each other, while groups with different letters are significantly different.)

Geographic Population	Burrowing Depth (cm) (Mean ± SD)	Burrowing Rate
Liaoning Dandong	3.92 ± 0.46 a	55%
Jiangsu Rudong	5.36 ± 0.36 b	40%
Guangxi Qinzhou	4.66 ± 0.51 c	65%

## Data Availability

All data from the study were used in the article.

## References

[1] Qi X. (2025). Research progress on the chemical composition, pharmacological activity, and quality control of the marine traditional Chinese medicine, scapharca subcrenata. Chin. J. Mar. Drugs.

[2] Wang C., Chai X., Wang H., Tang B., Liu B. (2013). Growth performance of the clam, *Meretrix meretrix*, breeding-selection populations cultured in different conditions. Acta Oceanol. Sin..

[3] Hua C.S., Hua C.A., Ping W.Y., Yu Z., Yi C. (2018). The effects of high temperatures on survival rates and immunity of saltwater clam *Meretrix meretrix* from different geographical populations. Isr. J. Aquac..

[4] Yasuo N., Tadashi N., Tatsuya Y., Yukio M., Takayoshi K., Akio T. (2010). Reproductive cycle of the venerid clam *Meretrix lusoria* in Ariake Sound and Tokyo Bay, Japan. Fish. Sci..

[5] Santos M.N.D., Gaspar M.B., Vasconcelos P., Monteiro C.C. (2002). Weight–length relationships for 50 selected fish species of the Algarve coast (southern Portugal). Fish. Res..

[6] Faal S.A., Esmaeili H.R., Teimori A., Shahhosseini G. (2025). Does fish scale morphology allow the identification of species complex? A case study for algae scraper fishes in Iran (Teleostei: Cyprinidae). Zool. Anz..

[7] Ping W.Y., Hua C.A., Xing Y.G., Ding S.H. (2010). The relationship between shell morphology and body weight of *Meretrix meretrix*. Mar. Fish..

[8] Wei L., Biao H.Q., Jie M., Ling F.J., Min M., Dong L.X. (2024). Differences in Morphology and Carbon and Nitrogen Contents of Venus Clam *Meretrix meretrix* from Different Geographical Populations. Fish. Sci..

[9] Hua C.A., Ping W.Y., Xing Y.G., Wei Z.Z., Ping W.J. (2010). Morphological variation analysis on *Meretrix lusoria* and 4 *Meretrix meretrix* populations. Mar. Fish..

[10] Deng Y., Du X., Huang R., Wang Q. (2008). Morphological and karyotypic variation in three wild populations of *Meretrix meretrix*. Chin. J. Oceanol. Limnol..

[11] Zhuang S.H., Wang Z.Q. (2004). Influence of size, habitat and food concentration on the feeding ecology of the bivalve, *Meretrix meretrix* Linnaeus. Aquaculture.

[12] Lei C., Yong G.L., Song Z., Kun X., Jian S., Qi L., Jie L.Y., Peng H.X., Fan C., Ying L.H. (2016). The observation and analysis of burrowing behaviors of ark shell Scapharca subcrenata in different substrates and sowing conditions. Fish. Mod..

[13] Jun H.G., Jie Z., Bin Y., Hua C.Y., Guo D.Z. (2024). Adaptability comparison of venus clam (*Cyclina sinensis*) with different sizes to three new sediments. J. Dalian Ocean Univ..

[14] Yuan J.Y., Qun C., Hong L.T., Ming S., Bin L.G., Wen H.F., Yi W.H., Wen Z.T., Guang H.F. (2024). Morphological Variation Analysis of Six Geographical Populations of Venus Clam *Meretrix meretrix*. Fish. Sci..

[15] Jun T.B., Zhong L.B., Sheng Y.H., Hai X.J. (2005). Oxygen consumption and ammonia-N excretion of *Meretrix meretrix* in different temperature and salinity. Chin. J. Oceanol. Limnol..

[16] Migenda N., Möller R., Schenck W. (2021). Adaptive dimensionality reduction for neural network-based online principal component analysis. PLoS ONE.

[17] Quwsar O.A., Firoz M.M., Benta S.F., Abdul H.M., Mostafa M.M. (2020). Autoembedder: A semi-supervised DNN embedding system for clustering. Knowl.-Based Syst..

[18] Qiong W., Han J.Y., Jian L., Tao L.J., Ying H.Y. (2024). Genetic structural analysis of different breeds and geographical groups of *Fenneropenaeus chinensis* reveals population diversity. Genomics.

[19] Chu M., Liu B., Ou L., Chen Z., Li Q. (2024). Morphological Differences and Contour Visualization of Statoliths in Different Geographic Populations of Purpleback Flying Squid (*Sthenoteuthis oualaniensis*). J. Mar. Sci. Eng..

[20] Richards C.S., Cruz D.V., Shapiro J.W., Wootton J.T., Pfister C.A. (2025). Spatially Varying Selection Amplifies Intrapopulation Differentiation Among Phenotypic Traits in the Rocky-Shore Mussel, *Mytilus californianus*. Ecol. Evol..

[21] Torres-Romero E.J., Morales-Castilla I., Olalla-Tárraga M.Á. (2016). Bergmann’s rule in the oceans? Temperature strongly correlates with global interspecific patterns of body size in marine mammals. Glob. Ecol. Biogeogr..

[22] Akester R.J., Martel A.L. (2000). Shell shape, dysodont tooth morphology, and hinge-ligament thickness in the bay mussel *Mytilus trossulus* correlate with wave exposure. Can. J. Zool..

[23] Hak L.J., Kim C.-H. (2013). Long-term variability of sea surface temperature in the East China Sea: A review. Ocean Polar Res..

[24] Ae P.K., Young L.E., Eunmi C., Sungwook H. (2015). Spatial and temporal variability of sea surface temperature and warming trends in the Yellow Sea. J. Mar. Syst..

[25] Yuan Z., Xiao X., Wang F., Xing L., Wang Z., Zhang H., Xiang R., Zhou L., Zhao M. (2018). Spatiotemporal temperature variations in the East China Sea shelf during the Holocene in response to surface circulation evolution. Quat. Int..

[26] Yue X.Q., Hong Z.J., Wu Y.X., Tao N.H. (2020). Genetic diversity and differentiation of nine populations of the hard clam (*Meretrix petechialis*) assessed by EST-derived microsatellites. Electron. J. Biotechnol..

[27] Kuo L., Tong L.X., Hao C.Z., Teng L., Lei F., Ming H.Z., Wu Y.X. (2021). Current trends in population research on shell morphological polymorphism of mollusks. Mar. Sci..

[28] Spooner D.E., Vaughn C.C. (2008). A trait-based approach to species’ roles in stream ecosystems: Climate change, community structure, and material cycling. Oecologia.

[29] Wang T., Li Q. (2018). Effects of salinity and temperature on growth and survival of juvenile Iwagaki oyster *Crassostrea nippona*. J. Ocean Univ. China.

[30] Simon P., Amber H., Mathews T.J. (2021). The effects of food quantity, light, and temperature on clearance rates in freshwater bivalves (Cyrenidae and Unionidae). Hydrobiologia.

[31] Muro R.L., Letts R.E. (2007). Responses of the mussel *Anodontites trapesialis* (Unionidae) to environmental stressors: Effect of pH, temperature and metals on filtration rate. Environ. Pollut..

[32] Jørgensen C., Larsen P., Riisgård H.U. (1990). Effect of temperature on the mussel pump. Mar. Ecol.-Prog. Ser..

[33] Yang H., Haili H., Dong L., Chengbo S., Zhigang L. (2013). Effects of body mass and temperature on oxygen consumption rate and ammonia excretion rate of *Soletellina acuta*. J. Trop. Biol..

[34] Tao N.H., Peng C., Ming H.Z., Yun C., Lin H.X., Feng Y., Wu Y.X. (2017). Effects of temperature and salinity on oxygen consumption and ammonia excretion in different colour strains of the Manila clam, *Ruditapes philippinarum*. Aquac. Res..

[35] Jorgensen C.B., Ockelmann K. (1991). Beat frequency of lateral cilia in intact filter feeding bivalves: Effect of temperature. Ophelia.

[36] Ding T., Bai Y., Nie H., Zhang Z., Yan X. (2019). Effects of Low Temperature and Low Salinity on Oxygen Consumption Rate and Ammonia Excretion Rate of Venus Clam *Meretrix meretrix*. Chin. J. Fish..

[37] Qing Z.Y., Hua C.S., Hua C.A., Dong Z.Z., Yu Z., Cao Y., Heng P.Y., Chao Z.Y., Xin Y.J., Ping W.Y. (2024). Effects of High Temperature Stress on the Survival, Metabolism, and Related Enzyme Activities of Two *Meretrix meretrix* Populations. Mar. Fish..

[38] Qi Z.M., Fei K., Bin M., Shui C.D., Shou R.Z., Nan M.S., Kai L., Yi C.J., Lin Z., Jun Y.X. (2023). Effects of light on growth, feeding rate, digestion, and antioxidation in juvenile razor clams *Sinonovacula constricta*. Aquaculture.

[39] Milan M., Carraro L., Fariselli P., Martino M.E., Duccio C., Vitali F., Boffo L., Patarnello T., Bargelloni L., Cardazzo B. (2018). Microbiota and environmental stress: How pollution affects microbial communities in Manila clams. Aquat. Toxicol..

[40] Akter S., Wos-Oxley M.L., Catalano S.R., Hassan M.M., Li X., Qin J.G., Oxley A.P.A. (2023). Host Species and Environment Shape the Gut Microbiota of Cohabiting Marine Bivalves. Microb. Ecol..

[41] Ming G.X., Cui L., Yan W.H., Zhe X. (2018). Diversity and Evolution of Living Oysters. J. Shellfish Res..

[42] Rahman M.A., Henderson S., Miller-Ezzy P., Li X.X., Qin J.G. (2019). Immune response to temperature stress in three bivalve species: Pacific oyster *Crassostrea gigas*, Mediterranean mussel *Mytilus galloprovincialis* and mud cockle *Katelysia rhytiphora*. Fish Shellfish Immunol..

[43] Li Y., Cheng Y., Chen K., Cheng Z., Zhu X., CRCardoso J., Liang X., Zhu Y., Power D., Yang J. (2020). Thyroid hormone receptor: A new player in epinephrine-induced larval metamorphosis of the hard-shelled mussel. Gen. Comp. Endocrinol..

[44] Lymbery A.J., Ma L., Lymbery S.J., Klunzinger M.W., Beatty S.J., Morgan D.L. (2020). Burrowing behavior protects a threatened freshwater mussel in drying rivers. Hydrobiologia.

[45] Carmo Reis G., Simeone D., Beasley C.R. (2023). Experimental evidence associates burrowing behavior of *Castalia ambigua* (Bivalvia: Hyriidae) with shell shape and density. Ecol. Evol..

[46] Sheng Z.C., Yan X.S., Qi L.J., Hui F.J., Lei L.L., Fei M.Z., Han Y.W., Nan Z.H., Ze M.Y. (2022). Influences of Substrate Grain Size on the Burrowing Behavior of Juvenile *Meretrix meretrix*. Animals.

[47] Feng Y., Chao Z., Hua W., Sheng W.Y., Yang H.Y., Yu Z. (2016). Effects of environmental factors and clam size on the burrowing behavior of Manila clam *Ruditapes philippinarum*. Acta Ecol. Sin..

[48] McLachlan A., Jaramillo E., Defeo O., Dugan J., Ruyck A., Coetzee P. (1995). Adaptations of bivalves to different beach types. J. Exp. Mar. Biol. Ecol..

[49] Shinji S., Yoichi W., Soonbo Y., Tomohiro K. (2011). Burrowing Criteria and Burrowing Mode Adjustment in Bivalves to Varying Geoenvironmental Conditions in Intertidal Flats and Beaches. PLoS ONE.

